# Paracellular Transport through Healthy and Cystic Fibrosis Bronchial Epithelial Cell Lines – Do We Have a Proper Model?

**DOI:** 10.1371/journal.pone.0100621

**Published:** 2014-06-19

**Authors:** Natalia Molenda, Katarina Urbanova, Nelly Weiser, Kristina Kusche-Vihrog, Dorothee Günzel, Hermann Schillers

**Affiliations:** 1 Institute of Physiology II, University of Münster, Münster, Germany; 2 Institute of Clinical Physiology, Charité Campus Benjamin Franklin, Berlin, Germany; University of Colorado, Denver, United States of America

## Abstract

It has been reported recently that the cystic fibrosis transmembrane conductance regulator (CFTR) besides transcellular chloride transport, also controls the paracellular permeability of bronchial epithelium. The aim of this study was to test whether overexpressing wtCFTR solely regulates paracellular permeability of cell monolayers. To answer this question we used a CFBE41o^–^ cell line transfected with wtCFTR or mutant F508del-CFTR and compered them with parental line and healthy 16HBE14o^–^ cells. Transepithelial electrical resistance (TER) and paracellular fluorescein flux were measured under control and CFTR-stimulating conditions. CFTR stimulation significant decreased TER in 16HBE14o^–^ and also in CFBE41o^–^ cells transfected with wtCFTR. In contrast, TER increased upon stimulation in CFBE41o^–^ cells and CFBE41o^–^ cells transfected with F508del-CFTR. Under non-stimulated conditions, all four cell lines had similar paracellular fluorescein flux. Stimulation increased only the paracellular permeability of the 16HBE14o^–^ cell monolayers. We observed that 16HBE14o^–^ cells were significantly smaller and showed a different structure of cell-cell contacts than CFBE41o^–^ and its overexpressing clones. Consequently, 16HBE14o^–^ cells have about 80% more cell-cell contacts through which electrical current and solutes can leak. Also tight junction protein composition is different in ‘healthy’ 16HBE14o^–^ cells compared to ‘cystic fibrosis’ CFBE41o^–^ cells. We found that claudin-3 expression was considerably stronger in 16HBE14o– cells than in the three CFBE41o^–^ cell clones and thus independent of the presence of functional CFTR. Together, CFBE41o^–^ cell line transfection with wtCFTR modifies transcellular conductance, but not the paracellular permeability. We conclude that CFTR overexpression is not sufficient to fully reconstitute transport in CF bronchial epithelium. Hence, it is not recommended to use those cell lines to study CFTR-dependent epithelial transport.

## Introduction

In the apical and basolateral membrane, embedded ion channels and transporters together provide for epithelial (transcellular) transport. The active transport is directly or indirectly ATP-dependent, while the passive one is driven by electrochemical gradients maintained by active transporters [1]. It is likely that the paracellular pathway is regulated in parallel with the transcellular pathway because both routes determine net transport and must work in concert as they are functionally matched to meet the transport requirements of a specific tissue [2]. On the apical membrane of epithelial cells localized cystic fibrosis transmembrane conductance regulator (CFTR) is a cyclic adenosine monophosphate (cAMP)-regulated channel, which is found in various organs like lung, pancreas, intestine, testes, and others [3], [4]. CFTR is a limiting factor of the airway epithelial fluid secretion and defect of this protein results in the impaired epithelial salt and water transport, causing stasis of mucus, chronic inflammation and infection in lung. Meanwhile, over 1,900 mutations of this protein are known (http://www.genet.sickkids.on.ca) and the most common mutation causing cystic fibrosis (CF) is the deletion of phenylalanine at position 508 (F508del) [5]. The CF phenotype is the consequence of CFTR insufficiency not only in terms of its chloride conductance but also concerning its regulatory function on other ion channels and intracellular interaction partners [6]–[8]. In this line, CFTR is assumed to be involved in the regulation of paracellular permeability [9]–[12]. Paracellular transport of solutes and water is driven by the transepithelial electrochemical gradient [13] and modulated by tight junctions (TJ), a multi-protein complex, which acts as a permeability barrier [14], [15]. Tight junctions allow paracellular permeation through at least two parallel pathways: i) a pore pathway - a system of charge-selective small pores (4 Å exclusion radius) and ii) a leak pathway - larger discontinuities in barrier, which lack charge and size discrimination [16]. The pore pathway has a high capacity and is responsible for the flux of specific ions and small uncharged solutes. However, through the leak pathway only a small amount of larger molecules can pass [17].

In the presented study, we compared polarized human bronchial epithelial cell line CFBE41o^–^ transfected with wild type CFTR (wtCFTR) and mutant F508del-CFTR [18] to 16HBE14o^–^ and CFBE41o^–^ cell lines, to investigate the influence of CFTR and F508del-CFTR on paracellular permeability. The commonly used 16HBE14o^–^ and CFBE41o^–^ cell lines have the disadvantage that they do not originate from the same donor and therefore they have a different genetic background. This potential problem can be solved by the overexpression of wtCFTRwtCFTR and F508del-CFTR in the CFBE41o^–^ cell line, which should mimic healthy and CF airway epithelia [18]. The aim of this study was to test if expression of wtCFTR in CF cells restores epithelial function, not only in terms of chloride conductance, but also regarding CFTR dependent regulation of paracellular permeability. Limiting for fluorescein flux (as a measure of paracellular solute transport) across epithelia is the protein structure and composition of TJ. Tight junction barrier function and charge selectivity are determined by claudins, a large family of integral tight junction transmembrane proteins [17]. Claudins and other TJ proteins are organized in a continuous network of parallel and interconnected strands at the lateral membranes of adjacent cells. However, permeability depends not only on microstructure regulated by molecular sieves formed by intermeshing TJ strands on the extracellular surface of opposing membranes but also on cell size [19]. Monolayers formed by small cells show more junctional length per area than large cells. Large junctional length per area means more paracellular shunts and subsequently, a higher transport capacity of these pathways. Together the transport capacity of the paracellular pathway is determined by TJ composition and cell morphology within a monolayer or a tissue. This was particularly considered in this study.

## Methods

### Materials

Unless specified otherwise, all chemicals were obtained from Sigma-Aldrich (Deisenhofen, Germany).

### Cell Culture

The cell line 16HBE14o^–^ was generated by the transformation of normal bronchial epithelial cells with SV40 large T-antigen [20]. The 16HBE14o^–^ cells retain differentiated epithelial morphology by forming polarized layers with microvilli and cilia [21]; [22]. The CFBE41o^–^ cell line was generated by transformation of cystic fibrosis (CF) tracheo-bronchial cells with SV40 and is homozygous for the F508del-CFTR mutation [23]. CFBE41o^–^ cell line was corrected with a plasmid containing either full-length (6.2kb) wild type wtCFTR (CFBE-WT) or F508del-CFTR (4.7 kb) (CFBE-delF) cDNA [18]. All cell lines were kind gifts from Dr. Dieter C. Gruenert, California Pacific Medical Center Research Institute, San Francisco, CA, USA. Cell lines were grown in Eagle’s Minimal Essential Medium (Invitrogen, Karlsruhe, Germany) and supplemented with 10% Fetal Bovine Serum (PAA Laboratories, Pasching, Austria), 2 mM L-glutamine, 50 U/ml penicillin and 50 µg/ml streptomycin. Additionally for CFBE41o^–^ cell lines transfected with plasmids 300 µg/ml Hygromycin B (InvivoGen, San Diego, CA, USA) was added as selective agent. All cell lines were cultured in a 5% CO_2_-95% air incubator at 37°C. The cell culture medium was changed three times per week. The confluent cells were trypsinized and 1.6×10^5^ cells were seeded on a ThinCert cell culture inserts (12-well plate, Greiner Bio-One, Germany). The flasks and inserts were coated with a solution containing collagen type I from calf skin and human plasma fibronectin (Invitrogen, Karlsruhe, Germany). In our experiments the numbers of subcultures (P) were P4.78 for CFBE41o^–^, P4.77.48 for CFBE-WT, P4.77.34 for CFBE-delF and P4.46 for 16HBE14o^–^ cells. The absolute number of passages (P) as denoted by “(passages after primary isolation).(passages after immortalization).(passages after CFTR transfection).

### Stimulation of Epithelial Cells

In order to activate CFTR related epithelial transport, cells were treated with 8-(4-chlorophenylthio) adenosine 3′, 5′cyclic monophosphate sodium salt (8cpt-cAMP) in final concentration of 100 µM.

### Continues Transepithelial Electric Resistance (cTER) Measurements

**Figure 1:**
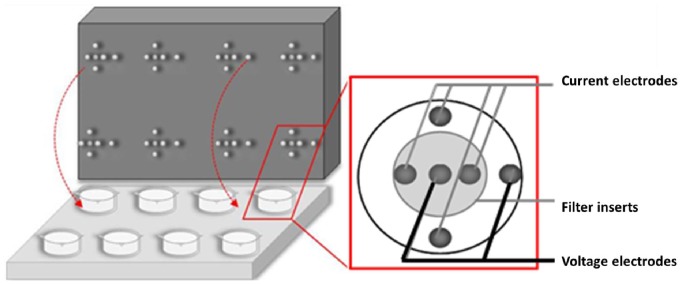
Scheme of the continuous transepithelial resistance measurement device (cTER). The ThinCert culture plate contains eight filter inserts with cell monolayers. The upper plate (lid) has six titanium electrodes for each insert, four electrodes to inject the current and two to measures the voltage. Electrodes are arranged in a way that resulted in a fairly homogenous electrical field.

Determination of cTER were performed as described previously [12]. Briefly, we used a cover lid for 12 well ThinCert plates equipped with 8 sets of six titanium electrodes (4 to inject current and two to measure voltage) creating a homogenous electrical field (Fig. 1). Electrical current pulses with a frequency of 125 Hz were applied at a given time interval for 1 s. The electrical resistance of cell-free ThinCert inserts (128 Ω·cm^2^) was subtracted from TER raw data. Data acquisition and processing was performed with a 2-channel PowerLab system (26 Series, ADInstruments GmbH, Germany).

### Ussing Chamber Experiments

For Ussing chamber experiments, 16HBE14o^–^ and CFBE41o^–^ cells were seeded on filter supports (Millicell-PCF, 0.4 µm pore size, Millipore) and grown to confluence within 7 days. Confluent cells layers on filter supports were directly mounted in conventional Ussing- chambers and both hemi-chambers were filled with 10 ml standard bath solution (in mM: 140 Na^+^, 123.8 Cl^−^, 5.4 K^+^, 1.2 Ca^2+^, 1.2 Mg^2+^, 2.4 HPO_4_^2−^, 0.6 H_2_PO_4_^−^, 21 HCO_3_^−^, 10 D(+)-glucose, pH 7.4 when equilibrated with 5% CO_2_ in O_2_ at 37°C). Flux measurements were carried out with and without applying voltage-clamp. After equilibration, flux tracers (fluorescein sodium salt, final concentration 100 µM) were added to the apical side and 300 µl aliquots were collected from the receiving side every 10 min and immediately replaced by fresh standard bath solution. Duplicates of fluorescein-containing samples were pipetted onto 96-well plates (140 µl/well) together with defined dilutions of fluorescein in standard bath saline for calibration and fluorescence was quantified using a plate reader (TECAN, infinite M200). Flux was calculated as increase in tracer quantity (corrected for dilution) per time unit and filter area (0.6 cm^2^).

### Immunostaining and Image Analysis

16HBE14o^–^, CFBE41o^–^ and CFBE41o^–^ cell lines transfected with wtCFTR or F508del-CFTR were grown to confluence on ThinCert inserts, fixed in 3,7% PFA at RT for 15 min, and then permeabilized with 0.1% Triton X-100 for 5 min. After blocking with 5% goat serum in PBS for 30 min, cells were incubated for 1 h with a mouse monoclonal antibody mouse-anti-ZO-1 (zonula occludens-1) (BD Transduction Laboratories, NJ, USA), mouse-anti claudin-4 and -5 or rabbit-anti caudin-3 and -7 (1∶200, Zymed/Invitrogen, San Francisco, CA) followed by labeled secondary antibody Alexa-FlourTM 488 goat anti-mouse (Molecular Probes, Inc., OR, USA) Cy2 goat anti-mouse or Cy5 goat anti-rabbit (1∶1000, Jackson Immuno Research, Newmarket, UK) and DAPI 1∶10000 (Invitrogen, OR, USA). Imaging was performed using a Zeiss 510 Meta laser scanning microscopy with a 63x oil objective or a Zeiss Observer Z fluorescence microscope with 40x and 100x oil objectives. Fluorescence micrographs were acquired using an EMCCD camera (iXon EM+ DU-888, Andor Technology, Belfast, Ireland) and MetaMorph (Molecular Devices, Inc., CA; USA).

The length and structure of the intercellular junction of monolayers were determined after staining of cells with antibodies against ZO-1 using ImageJ image analysis software (Rasband W.S., ImageJ, National Institutes of Health, Bethesda, Maryland, USA, http://rsb.info.nih.gov/ij/, 1997–2004).

### Measurement of Fluorescein Flux

The transport of the fluorescein sodium salt (fluorescein) was determined to investigate the paracellular permeability of cell monolayers grown on 12 well ThinCert inserts (all cell lines). Integrity of the monolayer was checked prior to the experiment by determination of TER. The cell monolayers were rinsed gently twice with MEM medium without phenol-red indicator and left for 1–2 hours to equilibrate in the same medium. The TER of the monolayers was measured again. A stock solution of fluorescein was mixed with fresh MEM and added to the apical compartment (end concentration 10 µM). Samples (100 µl) were withdrawn immediately and after 1 h from the apical and basolateral compartment. The flux of fluorescein across the cell layer was calculated from the net increase of the fluorescence in the basolateral compartment measured with a fluorescent microplate reader (Labsystem Fluoroskan II, GMI, Inc., USA) (E_x_ = 485 nm, E_m_ = 538 nm). Data are presented as the fluorescein flux (nmol h^−1^ cm^−2^).

### Western Blot

ProteoExtract Transmembrane Protein Extraction Kit (Calbiochem) was used for protein isolation according to the manufactures protocol ‘Extraction of Membrane Proteins from Adherent Tissue Culture Cells’. Protein concentration was determined by Pierce BCA assay and 10 µg protein per lane was loaded on 12.5% polyacrylamid gels. Subsequent to SDS gel electrophoresis, proteins were blotted onto polyscreen PVDF transfer membranes (Perkin Elmer, Waltham, MA). Blots were blocked in 5% BSA before overnight incubation with primary antibodies (mouse-anti claudin-4 and -5 or rabbit-anti caudin-3, -7 and -18, all Zymed/Invitrogen, 1∶1000). Peroxidase-conjugated AffiniPure F(ab′)_2_ fragment goat anti-mouse/−rabbit (1∶10000, Jackson ImmunoResearch) were used as secondary antibodies and Lumi-Light^PLUS^ Western Blotting Kit (Roche, Grenzach-Wyhlen, Germany) was used for visualizationin a Fusion FX 7 image acquisition system (Vilber Lourmat, Eberhardzell, Germany).

### RNA Extraction

All cell lines were grown on coated culture dishes. RNA was extracted from confluent cells using the RNeasy mini kit (Qiagen Hilden, Germany). The RNA was DNase treated (Deoxyribonuclease I, Invitrogen, Karlsruhe, Germany). The total RNA concentration and quantity were assessed by the absorbance at 260 nm using a Bio Photometer (Eppendorf, Hamburg, Germany). 1 µg of total RNA was reverse-transcribed to cDNA using SuperScript III Reverse Transcriptase (Invitrogen, Karlsruhe, Germany). For each cell line three cDNA samples from separate RNA extractions and reverse transcription reactions were employed.

### Real-time PCR

Quantitative analysis of the DNA and RNA was performed in 20 µl with 1 µM each of predesigned TaqMan Gene Expression Assays (Applied Biosystems, Foster City, CA, USA) in Eppendorf Mastercycler ep realplex^4^ PCR thermal cycler (Eppendorf, Hamburg, Germany). The assay ID numbers of the validated genes are as follows: Hs00357011-m1 for CFTR and Hs02758991-g1 for GAPDH (Applied Biosystems, Foster City, CA, USA). The amplification was performed as follows: an initial step at 95°C for 10 min, followed by 45 cycles of 95°C for 15 sec and 60°C for 60 sec. All samples were run in triplicate. The 2^−ΔΔCT^ method was used to calculate the amount of gene expression [24]. CFTR mRNA expression was normalized to the parallel measured endogenous controls GADPH in each cell line. CFTR expression value of 16HBE14o^–^ cells was defined as 1 and CFTR expression of all other cells were normalized to this value.

### Statistics

Median values ± quartiles or mean ± SD are reported here. The Mann–Whitney–Wilcoxon (MWW) tests, One-way Anova analysis or Student t test were performed, respectively, to test for statistical significance. A p-value of <0.05 was accepted to indicate significant differences.

## Results

### Continous Transepithelial Electrical Resistance (cTER)

**Figure 2:**
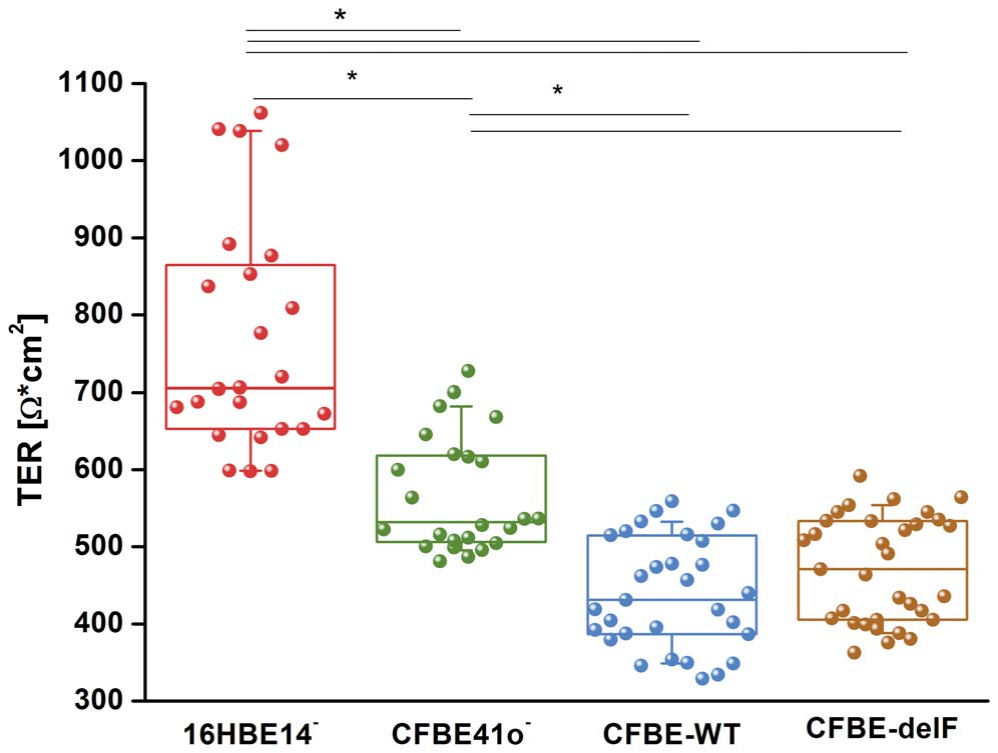
Continuous Transepithelial electrical resistance measurement (cTER). All cell monolayer were grown on ThinCert supports. cTER was monitored in all cell lines after they had reached confluence. 16HBE14o^–^ cells exhibit significantly higher TER values in comparison to CFBE41o^–^ and its transfected clones. Data are presented as a box-plots showing raw data (circles), median (horizontal line), 25 and 75 percentile (box) and SD (whiskers) (n = 24–33, p<0.05).

Epithelial cell monolayers can be characterised by cTER, which is determined primarily by the electrical resistance of the apical and basolateral cell membrane and the paracellular resistant, generated by the TJ complex [25]. A high TER value suggests a low permeability to ionic movement. TER values of 24 to 33 independent experiments showed rather large variations. Therefore, data are expressed as median [first quartile; third quartile]. Median electrical resistances of monolayers after 8–11 days in culture was 703 [854; 651] Ω*cm^2^ for 16HBE14o^–^ and 528 [616; 504] Ω*cm^2^ for CFBE41o^–^ after correction for the background contributed by the ThinCert insert and medium (Fig. 2). 16HBE14o^–^ showed statistically significant higher TER than CFBE41o^–^. The TER of CFBE-delF cell (470 [534; 407] Ω*cm^2^) and CFBE-WT cell monolayers (431 [514; 387] Ω*cm^2^) were comparable to TER of the parental CFBE41o^–^ cell line (no statistical significance).

**Figure 3:**
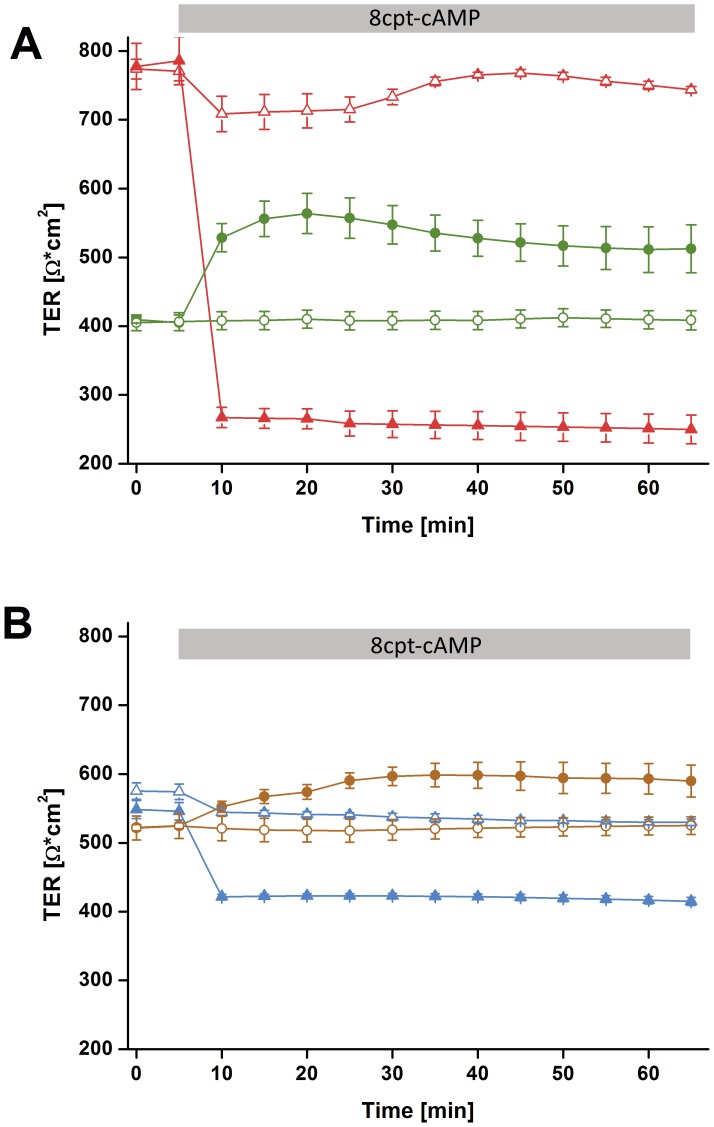
Changes in transepithelial electrical resistance (cTER) upon cAMP. **A)** 16HBE14o^–^ (triangles) and CFBE41o^–^ (circles) monolayer were grown on ThinCert supports. After 8–11 days in culture they obtained 800 and 500 Ω*cm^2^ resistance respectively. After 5 min 8cpt-cAMP was added, causing dramatic TER decrease in 16HBE14o^–^ cells (red triangles) and an increase of TER for CFBE41o^–^ (green circles). Addition of the same amount of medium to control cells (open circles and triangles) did not show any effect. **B)** CFBE-WT monolayer (blue triangles) and CFBE-delF monolayer (brown circles) were grown on ThinCert supports. After 8–11 days in culture both clones obtained 500–600 Ω*cm^2^ resistance. Stimulation with 8cpt-cAMP caused a decrease of TER in CFBE-WT cells (blue triangles) and an increase for CFBE-delF (brown circles). Addition of the same amount of medium to control cells (open circles and triangles) did not show any effect. Results are presented as mean ± SD (n = 3–6, p<0.05).

Stimulation of CFTR with cAMP resulted in a breakdown of resistance in all wtCFTR expressing cell monolayers. TER of 16HBE14o^–^ cells decreased by 60% within 5 minutes (Fig. 3A) and also CFBE-WT cell monolayer shows a clear but weaker response upon CFTR stimulation (decrease of TER by 30% within 5 minutes, Fig. 3B). By contrast, TER of all F508del-CFTR expressing cells rose transiently. CFBE41o^–^ increased by 30% and CFBE-delF by 10% respectively after stimulation with cAMP (Fig. 3A, B).

### Fluorescein Flux

**Figure 4:**
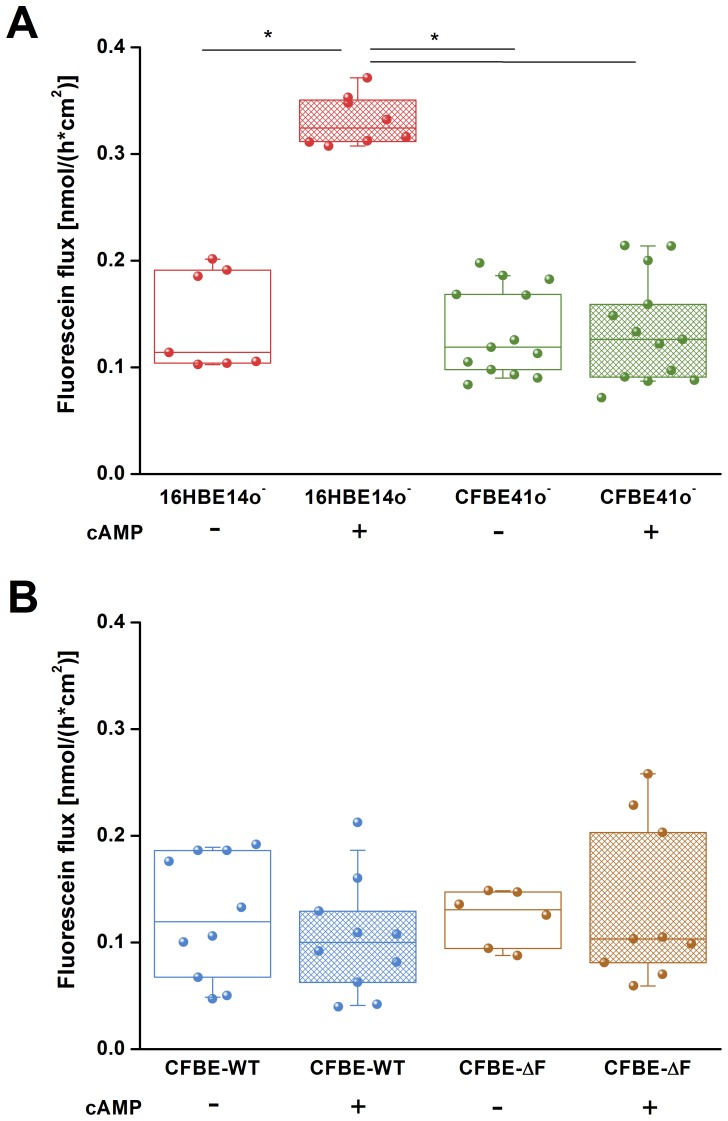
Paracellular permeability to fluorescein. **A)** Under control conditions 16HBE14o^–^ (red) and CFBE41o^–^ (green) cell lines showed a comparable fluorescein flux. Addition of cAMP significantly increased fluorescein flux of 16HB14o^–^ (red hatched box) but it did not affect CFBE41o^–^ cells (green hatched box). **B)** CFBE-WT cells (blue) and CFBE-delF (brown) do not show statistically significant differences in fluorescein flux neither under resting conditions nor upon stimulation with cAMP (hatched boxes). Data are presented as a box-plot showing raw data (circles), median (horizontal line) 25 and 75 percentile (box) and SD (whiskers) (n = 7–13, p<0.05).

To distinguish between the transcellular und paracellular permeability we measured the flux of the tracer molecule fluorescein, which indicates the movement exclusively through the paracellular pathway [26]. Under control conditions fluorescein flux was comparable in all tested cell lines: 0.114 [0.191; 0.103] nmol *cm^−2^ h^−1^ for 16HB14o^–^, 0.118 [0.167; 0.098] nmol *cm^−2^ h^−1^ for CFBE41o^–^, 0.105 [0.185; 0.066] nmol *cm^−2^ h^−1^ for CFBE-WT and 0.125 [0.147; 0.094] nmol *cm^−2^ h^−1^ for CFBE-delF (Fig. 4A, B). Stimulation of CFTR with cAMP significantly increased fluorescein flux across the 16HBE14o^–^ monolayers (0.317 [0.348; 0.311] nmol *cm^−2^ h^−1^) but did not affect flux across CFBE41o^–^ (0.125 [0.158; 0.091] nmol *cm^−2^ h^−1^) (Fig. 4A), CFBE-WT (0.091 [0.128; 0.062] nmol *cm^−2^ h^−1^) and CFBE-delF (0.103 [0.203; 0.081] nmol *cm^−2^ h^−1^) monolayers (Fig. 4B). These results indicate that stimulation of CFTR affects only paracellular permeability of healthy epithelia (16HBE14o^–^).

Because fluorescein is a negatively charged molecule, we tested if the transepithelial potential difference influenced the paracellular flux of fluorescein. First, there was no preferential direction of fluorescein transport across 16HBE14o^–^ cell monolayers (apical to basolateral or vice-versa). Second, there was no difference in fluorescein transport in Ussing-chamber experiments when transepithelial potential difference was clamped to zero by injecting short-circuit current. The permeability to fluorescein in 16HBE14o^–^ cell monolayers exposed to voltage clamp shows no statistically significant difference to 16HBE14o^–^ cells without voltage clamp (n = 13). We observed the same effects for CFBE41o^–^ cells (data not shown).

### Cell Morphology

The paracellular flux depends not only on TJ structure and composition but also on junctional length (cell-cell contact length) per area. Monolayers formed by small cells show usually more junctional length per area than large cells. But junctional length depends not only on cell size but also on microstructure of cell-cell contacts (wrinkles, folds, grooves, etc.). More junctional length per area means higher transport capacity due to more paracellular shunts per area (assuming a comparable TJ protein composition). In summary the transport capacity of the paracellular pathway is determined by TJ composition and cell morphology within a monolayer or a tissue.

**Figure 5:**
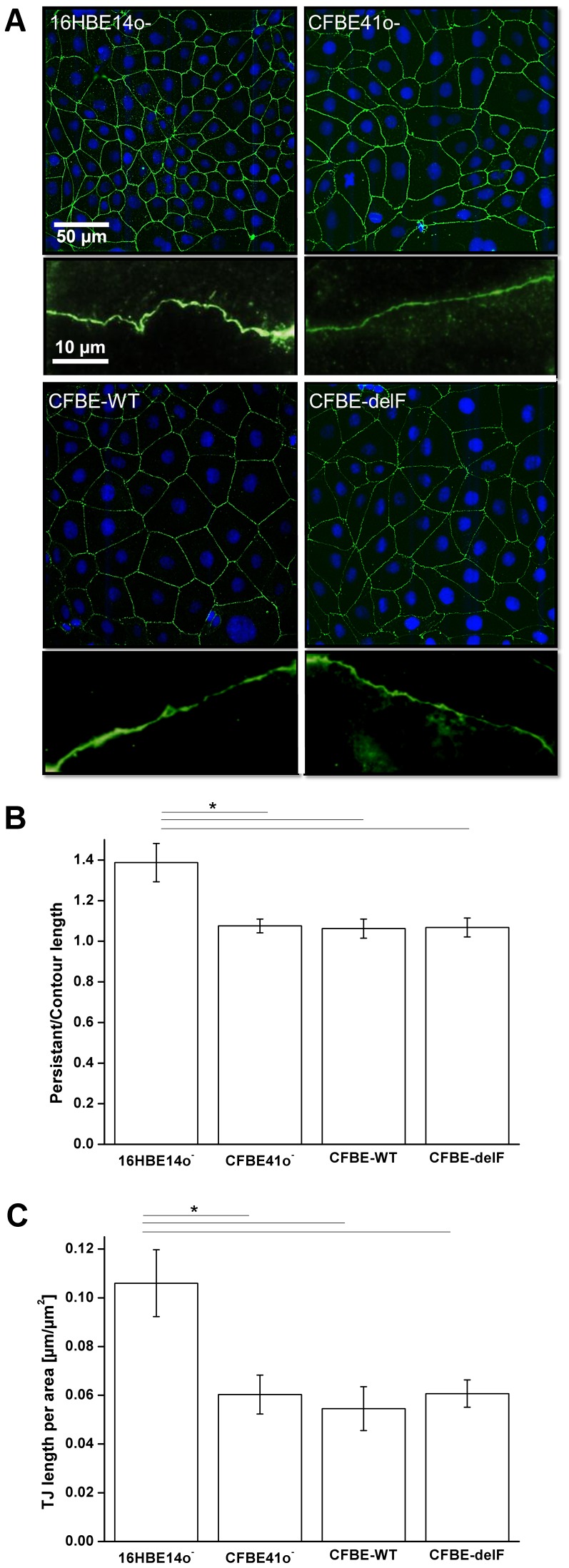
Differences in TJ length. **A)** Upper image show immunostaining of ZO-1 (green) and nuclei staining (blue). Lower image shows magnifications of representative cell borders. **B)** Ratio of persistent length to contour length of all tested cell monolayers. 16HBE14o^–^ shows a cell-cell contact enlargement by 40% while CFBE41o^–^ cells and its transfected clones exhibit only a tiny enlargement by 10%. **C)** TJ lengths per area in µm per µm^2^. 16HBE14o^–^ cells show 80% longer TJ lengths per area than CFBE41o^–^ cells. Furthermore, there is no difference in TJ lengths per area between CFBE41o^–^ cells and its transfected clones. Results are presented as mean ± SD (n = 7–8, p<0.05).

**Figure 6:**
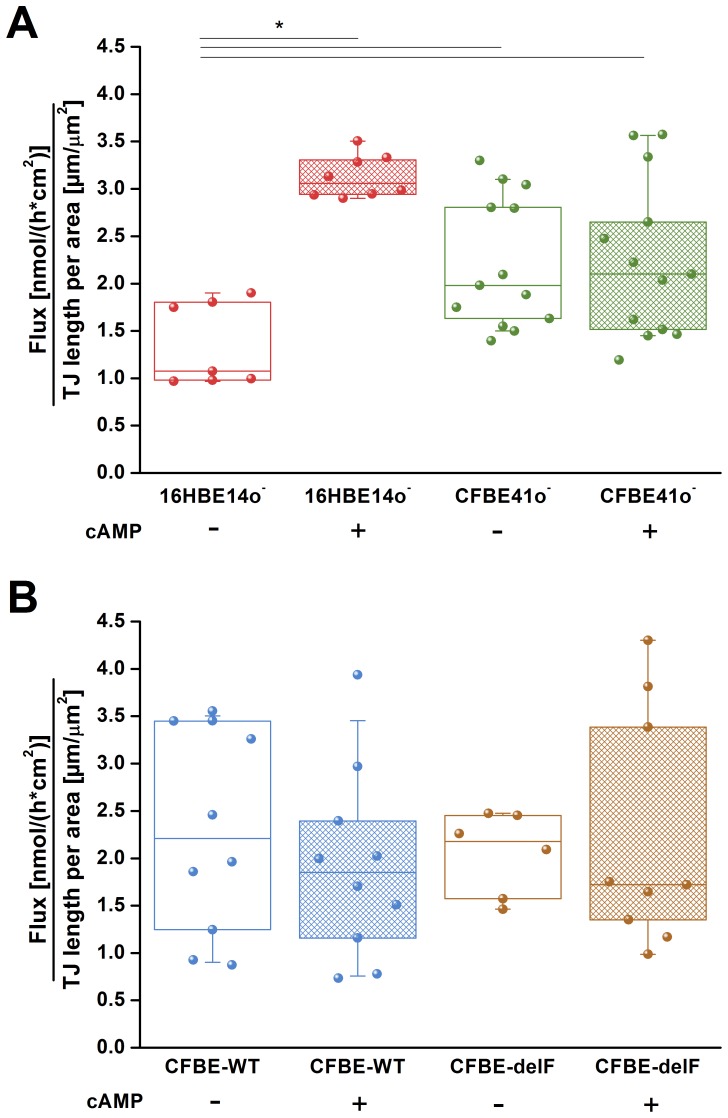
Fluorescein flux per TJ length. **A)** Calculation of flux per TJ length revealed a significant lower value for 16HBE14o^–^ cells under resting conditions (red box) compared to CFBE41o^–^ cells (green box). Stimulation with cAMP caused a 3 fold increase for 16HBE14o^–^ cells (red hatched box) while CFBE41o^–^ cells do not respond to stimulation (green hatched box). **B)** CFBE-WT cells (blue) and CFBE-delF (brown) do not show statistically significant differences in fluorescein flux per TJ length neither under resting conditions nor upon stimulation with cAMP (hatched boxes). Data are presented as a box-plot showing raw data (circles), median (horizontal line) 25 and 75 percentile (box) and SD (whiskers (n = 7–13, p<0.05).

Morphology of TJ pathway plays an important role in paracellular permeability. TJ length per area was derived from monolayers immunolabeled for a marker of tight junctions (ZO-1) (Fig. 5A) and analysed by imaging software ImageJ. Length of 16HBE14o^–^ TJ per µm^2^ is about 30% higher compared to CFBE41o^–^ and its transfected clones (Fig. 5C), so they have more junctional length per unit area through which electrical current and solutes can pass. 16HBE14o^–^ cells have also more branched cell borders than the other cell lines. The ratio of persistent length to contour length is about 1.4. In contrast CFBE41o^–^ cells show a ratio of about 1.1 (Fig. 5B). Considering these ratios allow calculating the TJ length per µm^2^ for each cell line. As a result, 16HBE14o^–^ cells show about 80% higher length of cell-cell contact per area than CFBE41o^–^ and its transfected clones (Fig. 5C). Assuming the same TJ composition, a monolayer with smaller cells should have lower TER and higher solute flux [16]. However we observed that 16HBE14o^–^ monolayers show significantly higher TER values than CFBE41o^–^ and CFBE41o^–^ clones transfected with wtCFTR or F508del-CFTR. Epithelial permeability to fluorescein, a measure of passive leakage across the epithelium was correlated to the TJ length per area (Fig. 6A, B). All cell lines show statistically indistinguishable fluorescein flux under control conditions.

### Claudin Expression

**Figure 7:**
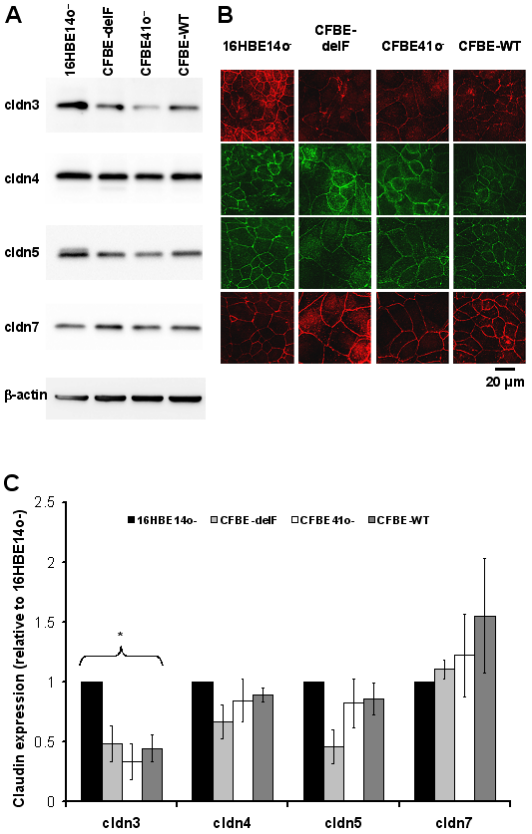
Detection of tight junction proteins. Presence of tight junction proteins known to be expressed in alveolar epithelial cells (claudin-3, -4, -5, -7) and their junctional localization in 16HBE14o^–^, CFBE41o^–^ cells and its transfected clones was verified by Western blot (A) and confocal laser scanning microscopy (B). For densitometric evaluation of Western blots (C), all signals were normalized to β-actin. All values are expressed relative to the respective value detected in 16HBE14o^–^ cell layers. One-way Anova analysis revealed that claudin-3 expression differed (p<0.05) in 16HBE14o^–^ and CFBE41o^–^ clones, whereas claudin-4, -5 and -7 expression was not significantly different (n = 4). No claudin-18 expression was detected (not shown).

Based on these results it is likely that the TJ composition differs between the investigated cell lines. Both Western blot and immuno-stainings demonstrated that 16HBE14o^–^ and CFBE41o^–^ cells expressed claudins-3, -4, -5 and -7 that had previously been described to be highly expressed in alveolar epithelial cells (Fig. 7, for rev. see [27]). No specific staining for claudin-18 could be detected (date not shown). Whereas expression for claudin-4, -5 and -7 was comparable for 16HBE14o^–^ and all CFBE41o^–^ cell clones, claudin-3 expression was considerably stronger in 16HBE14o^–^ cells than in the CFBE41o^–^ cell line and its clones, and thus independent of the presence of functional CFTR. Claudin-3 has a tightening effect on the paracellular pathway [28]. Results demonstrated that the TJ composition of 16HBE14o^–^ and CFBE41o^–^ cells is clearly different whereas CFBE41o^–^ exhibits a claudin expression, which is comparable to its clones transfected with wtCFTR or F508del-CFTR.

### Analysis of CFTR mRNA Expression by Real-time PCR

**Figure 8:**
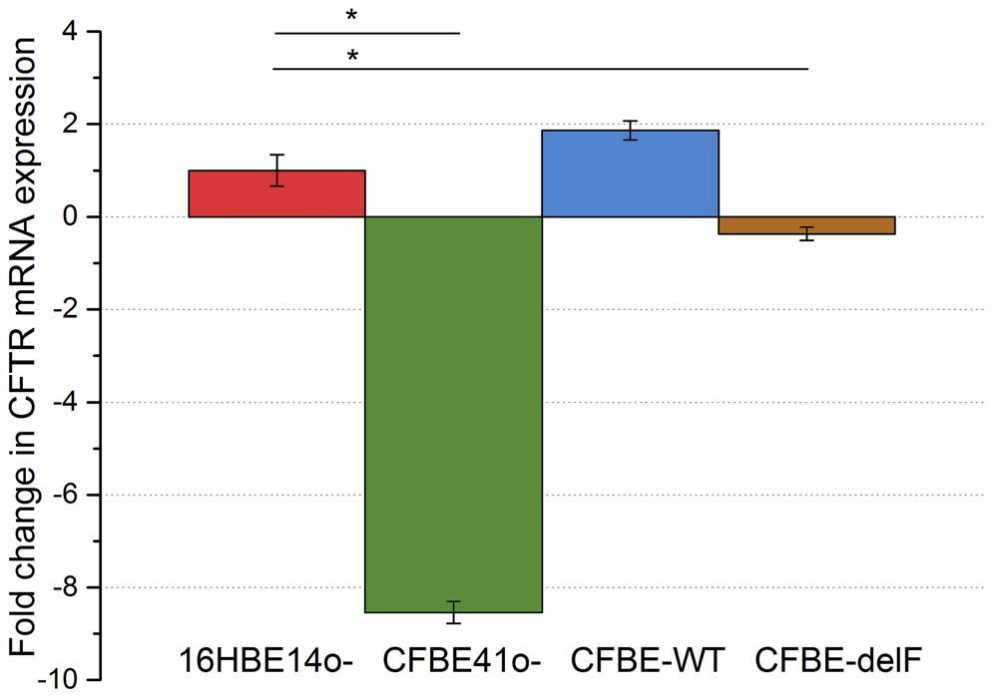
Expression of CFTR mRNA. The relative quantity of CFTR gene expression was calculated by the 2^−ΔΔCt^ method, using GAPDH as the internal reference. CFTR mRNA expression value of 16HBE14o^–^ cells was defined as 1 and expression of all other cells were normalized to this value. CFTR mRNA levels of the other cell lines were displayed as a fold change relative to16HBE14o^–^. Results are presented as mean ± SEM (n = 9–12, p<0.001).

The levels of CFTR mRNA in all cell lines were quantified by real-time PCR. CFTR expression value of 16HBE14o^–^ cells was defined as 1 and CFTR expression of all other cells were normalized to this value. CFTR mRNA levels of the other cell lines were displayed as a fold change relative to16HBE14o^–^ (Fig. 8). Vector-driven wtCFTR mRNA level in the complemented CFBE41o- clone (CFBE-WT) was 1,864 fold and not significantly higher compared to native CFTR mRNA in 16HBE14o- cells. CFBE-delF cells show an even lower mRNA expression (−0,37 fold, Fig. 8). Lowest expression of CFTR mRNA was found in CFBE41o- cells.

## Discussion

We previously compared paracellular permeability of 16HBE14o^–^ and CFBE41o^–^ cell lines [12]. Both of them represent widely used models of the human healthy and CF bronchial epithelium. We could not completely exclude that the differences between those two cell lines originated from the fact that they came from different donors instead of different CFTR expression in cell membrane. Thus, we tested if the same relationships of TER and fluorescein flux could be observed between the CFBE-WT and CFBE-delF cells. Those clones are new models of healthy and CF epithelium, created by transfection with wtCFTR or F508del-CFTR in the parental CFBE41o^–^ cell line [18]. Under control conditions 16HBE14o^–^ showed significantly higher TER values than CFBE41o^–^ and its clones (CFBE-WT and CFBE-delF). TER of both clones is indistinguishable but significantly lower that TER of their parental cell line (CFBE41o^–^) (Fig. 2). These results are consistent with the observations of Nilsson et al., who found that CFTR overexpression in CFBE41o^–^ cells (CFBE-WT) cells reduces TER [29]. However, it was also reported that transient and stable CFTR expression in CFBE41o^–^ cells increased TER [30]. Furthermore, Illek et al. demonstrated by Ussing chambers experiments that monolayers of parental CFBE41o^–^ and both transfected clones showed similar, moderately “tight” transepithelial resistance [18]. Though, the methodology, used by these research groups was not consistent. LeSimple et al. used chop stick electrodes with an epithelial voltmeter (EVOM) [30] and Nilsson et al. an ENDOHM-6 Chamber with an EVOM [29]. Chop-stick electrodes are known for spatial constrains showing an unequal distribution of current flow through the epithelium. It is conceivable that the divergence of results is a consequence of different methods. To overcome this difficulty, we developed a six-electrode system creating a homogenous electrical field (Fig. 1) and applied electrical current pulses with a frequency of 125 Hz.

Stimulation of CFTR with cAMP resulted in the decrease of TER in both wtCFTR expressing cell lines (Fig. 3A, B). The decrease of TER by 60% and 30% in 16HBE14o^–^ and CFBE-WT cells, respectively, after CFTR stimulation was most likely caused by the increase of membrane permeability due to the activation of the CFTR chloride channel and other CFTR dependent membrane conductance [31]. In CFBE41o^–^ and CFBE-delF cells application of cAMP showed an opposite effect, TER increased by 30% and 10%, respectively (Fig. 3A, B). Also Nilsson et al. observed the same acute effect on TER by 16HBE14o^–^ and CFBE-WT cells treated with cAMP raising agents: forskolin or IBMX [29]. Obviously the second messenger cAMP induced a change in TER. Even if the mechanism is unclear, the effect on TER is linked to the presence of functional CFTR. The responses of CFBE-WT and CFBE-delF cells were clearly weaker than by 16HBE14o^–^ and CFBE41o^–^, respectively. Illek et al. suggested that CFTR expression from the transgene is not as effective at generating working channels as the CFTR endogenously expressed in 16HBE14o^–^ cells. This way they explained why cells overexpressing CFTR at mRNA level were not showing same response to CFTR stimulation as 16HBE14o^–^ cells [18]. Illek et al. generated the cell lines CFBE-WT and CFBE-delF and measured their CFTR mRNA levels shortly after stable transfection (p4.77.05–p4.77.12). The expression of CFTR mRNA in the complemented cell lines was 14 to 27-fold higher (determined relative to 16HBE14o^–^). In contrast we showed that CFBE-WT do not have significantly higher CFTR expression than 16HBE14o^−^ cells (Fig. 8). Additionally in CFBE-delF mRNA expression was significantly lower than in 16HBE14o^–^ cells. One explanation could be that phenotypic stability is threatened by genetic and epigenetic changes, cell aging, and differentiation [32]. As a consequence, protein of interest expression can be reduced with time. In our experiments the numbers of subcultures (P) were P4.77.48 for CFBE-WT and P4.77.34 for CFBE-delF cells. Younger clones (lower number of P) are not available. Even Illek et al. performed measurements of transepithelial resistance and ion transport in Ussing chambers using cells p4.77.47 to p4.77.52 for CFBE-WT and p4.72.44 to p4.72.49 for CFBE-delF. Obviously the vector-driven CFTR expression in the available complemented cell lines (CFBE-WT and CFBE-delF) is not sufficient to mimic CFTR function in epithelial transport. CFTR is a key molecule for transepithelial transport which is not only determined by transcellular, but also by paracellular permeabilities and both permeabilities have to be functionally matched to comply with transport requirements of airway epithelia [2]. In a previous work, we showed with ^14^C-mannitol flux that stimulation of 16HBE14o^–^ cells (endogenously expressing wtCFTR) with cAMP increase their paracellular permeability [12]. Here, we tested the paracellular permeability of 16HBE14o^–^, CFBE41o^–^ and its transfected clones with the marker molecule fluorescein. Fluorescein sodium salt is a fluorescent, low molecular weight compound (radius 4.5 Å) and it can be used as a marker of paracellular permeability. We performed Ussing-chamber experiments with or without applying short-circuit current to eliminate electrical potential across the epithelium. Although fluorescein has negative charge, there was no change in permeability to fluorescein from apical side to basolateral side or vice-versa. From these data, we assume that the transepithelial potential difference does not influence fluorescein flux. This is consistent with results of Koljonen et al, who used fluorescein as an alternative marker molecule for mannitol in the studies of paracellular absorption and leakage in the apical – basolateral direction in Caco-2 cell monolayers [26]. Sodium fluorescein seems to be transported actively when a pH gradient is applied between the apical and the basolateral compartment [33], [34]. Therefore, we carried out all experiments under iso-pH conditions of pH 7.4 on both sides of the cell monolayer. We found that under control conditions, the fluorescein permeability of 16HBE14o^–^, CFBE41o^–^, CFBE-WT and CFBE-delF was statistically indistinguishable (Fig. 4A, B). cAMP stimulation significantly enhanced the paracellular permeability of 16HBE14o^–^ cell monolayers, but no changes in fluorescein flux were observed in CFBE41o^–^, CFBE-WT and CFBE-delF cells. These data demonstrate that expression of wtCFTR in CFBE41o^–^ is not sufficient to restore CFTR’s regulatory function on paracellular transport.

To explain those discrepancies we investigated structure and morphology of cell-cell contacts. The length of cell-cell contacts per area reflects the density of paracellular pathways within a cell monolayer. Immunostaining of the TJ protein ZO-1 revealed that 16HBE14o^–^ cells are considerably smaller than CFBE41o^–^, CFBE-WT and CFBE-delF cells. Transfection with wtCFTR (CFBE-WT) or F508del-CFTR (CFBE-delF) did not influence the morphology of the cells. The smaller 16HBE14o^–^ cells show clearly longer cell-cell contacts per area than the larger CFBE41o^–^ cells and its transfected clones (Fig. 5C). Furthermore, the microstructure of 16HBE14o^–^ cell-cell contacts shows wrinkles and ruffles, while CFBE41o^–^ and its transfected clones have smooth and wrinkle-free cell borders. This structural difference could be expressed as a ratio of persistent length over contour length. Contour length is roughly the circumference of the cell while persistent length describes the real length of cell-cell contacts, enlarged by wrinkles and ruffles. A ratio of persistent length over contour length of one means that there is no enlargement of cell-cell contacts and ratio over one indicate an enlargement of cell-cell contact length by infoldings and grooves. Figure 5B shows clearly the enlargement of cell-cell contact length for 16HBE14o^–^ cell monolayers (about 40%). In contrast, the ratio for CFBE41o^–^ and its transfected clones is close to one. Together, difference of TJ length per area is further increased by the microstructure of cell-cell contacts and is two-fold larger for 16HBE14o^–^ cell monolayers than for CFBE41o^–^ and its transfected clones (Fig. 5A). If the protein composition of TJ were comparable in all four tested cell lines, 16HBE14o^–^ cells should show lowest TER and highest paracellular permeability under control conditions. Actually, the TER of 16HBE14o^–^ cells was significantly higher than those of CFBE41o^–^ and its transfected clones (Fig. 2). We could show that fluorescein flux of all cell lines was statistically indistinguishable under control conditions (Fig. 4). However, when taking into consideration the difference in TJ length, fluorescein flux per unit of 16HBE14o^–^ cells was significantly lower under control conditions and significantly higher under CFTR-stimulated conditions (Fig. 6A). The fluorescein flux of CFBE41o^–^ was not influenced by cAMP and did not show differences to those of CFBE-WT and CFBE-delF (Fig. 6A, B).

In addition to difference in size and subsequently cell-cell contact length per area, we also demonstrated differences in TJ composition between 16HBE14o^–^ cells and CFBE41o^–^ and CFBE41o^–^ clones transfected with wtCFTR or F508del-CFTR. TJ consist of many membrane and scaffolding proteins and paracellular permeability of cells depends mostly on the claudin profile. There are 27 claudins known in mammals and differences in their expression are responsible for changes in the electrolyte and solute permeability in cells layers [35]. Even subtle changes in the structure of the TJ could profoundly affect the overall permeability or the ion selectivity of an epithelium [36] [2]. We discovered significantly higher membrane expression levels of the claudin-3 in 16HBE14o^–^ in comparison with other cells (Fig.7). This could explain the observed leak tightness of this cell line. In agreement with our results, elevating claudin-3 expression of MDCK cells caused strong increased in the paracellular resistance [28]. Recently, Lin et al. demonstrated that knockdown of the expression of either claudin 3 or 4 in ovarian carcinoma cells increased cell size and resulted in flattened morphology [37]. Cell lines investigated in this work show the same effect, since claudin 3 expression correlates with cell morphology (Fig. 5A, Fig. 7).

Nilsson et al. demonstrated that TJ integrity of 16HBE14o^–^, CFBE41o^–^ and CFBE transfected with wtCFTR cells was reduced upon CFTR stimulation with forskolin and IBMX [29]. This suggests a possible interaction between CFTR and the TJ protein complex, probably via the cytoskeleton. Also LeSimple [30] found that CFTR trafficking but not CFTR channel function is required for proper function and organization of TJs. We observed that increasing the intracellular concentration of cAMP increases paracelluar flux of fluorescein in the 16HBE14o^–^ cells. This effect depends not solely on CFTR because even transfection with wtCFTR could not restore the ability of CFBE41o^–^ cells to regulate paracellular permeability (Fig. 6A, B). Obviously, it is not only the missing CFTR chloride conductance which distinguishes a healthy from a CF epithelial cell.

In summary, we found that 16HBE14o^–^ cells have about 80% more junctional length per area than CFBE41o^–^ and its transfected clones and two-fold higher claudin 3 expression. This alone is enough to explain differences in paracellular transport between 16HBE14o^–^ and CFBE41o^–^ clones. We observed that transfection with wtCFTR in CFBE41o^–^ cell line restores transcellular, but not the paracellular conductance. We concluded that transfection with wtCFTR in CFBE41o^–^ cells (CFBE-WT) does not reconstitute healthy epithelium. Additionally this transfection is not as stable as originally reported [18]. Hence, cell lines investigated in this work are not suitable to study CFTR dependent epithelial transport.
